# Correction: Aktar et al. Gene Expression Analysis of Immune Regulatory Genes in Circulating Tumour Cells and Peripheral Blood Mononuclear Cells in Patients with Colorectal Carcinoma. *Int. J. Mol. Sci.* 2023, *24*, 5051

**DOI:** 10.3390/ijms241411362

**Published:** 2023-07-12

**Authors:** Sharmin Aktar, Faysal Bin Hamid, Sujani Madhurika Kodagoda Gamage, Tracie Cheng, Nahal Pakneshan, Cu Tai Lu, Farhadul Islam, Vinod Gopalan, Alfred King-yin Lam

**Affiliations:** 1 Cancer Molecular Pathology, School of Medicine and Dentistry, Menzies Health Institute Queensland, Griffith University, Gold Coast, QLD 4222, Australia; sharmin.aktar@griffithuni.edu.au (S.A.); faysal-bin.hamid@alumni.griffithuni.edu.au (F.B.H.); skodagod@bond.edu.au (S.M.K.G.); tracie.cheng@griffith.edu.au (T.C.); nahal.pakneshan@yahoo.com (N.P.); cutlu01@yahoo.com (C.T.L.); 2 Department of Biochemistry and Molecular Biology, Mawlana Bhashani Science and Technology University, Tangail 1902, Bangladesh; 3 Faculty of Health Sciences & Medicine, Bond University, Gold Coast, QLD 4229, Australia; 4 Department of Surgery, Gold Coast University Hospital, Gold Coast, QLD 4215, Australia; 5 Department of Biochemistry and Molecular Biology, University of Rajshahi, Rajshahi 6205, Bangladesh; farhad_bio83@ru.ac.bd; 6 Pathology Queensland, Gold Coast University Hospital, Southport, QLD 4215, Australia

Faysal Bin Hamid was not included as an author in the original publication [1]. F.B.H. actively contributed to this project. This study is an ongoing project, and he has helped with sample collection. He also contributed significantly to the validation of methods utilised in this study, particularly the CTC enrichment technique. The corrected Author Contributions Statement appears here. The authors apologise for any inconvenience caused and state that the scientific conclusions are unaffected. This correction was approved by the Academic Editor. The original publication has also been updated.

**Author Contributions:** Conceptualization, methodology, investigation, data interpretation, and writing—original draft preparation: S.A.; sample collection, methodology, and validation: F.B.H.; data acquisition: S.M.K.G., T.C., N.P. and C.T.L.; supervision, conceptualization, and writing—review and editing: F.I., V.G. and A.K.-y.L. All authors have read and agreed to the published version of the manuscript.
