# Supplementary material for: Gene Expression Analysis of Immune Regulatory Genes in Circulating Tumour Cells and Peripheral Blood Mononuclear Cells in Patients with Colorectal Carcinoma
Source: Int J Mol Sci. 2023 Mar 6;24(5):5051. doi: 10.3390/ijms24055051 (PMC10003441; doi:10.3390/ijms24055051)
Supplement: Supplementary file 1 [file ijms-24-05051-s001.zip › ijms-2200458 Table S1.pdf]

Table S1. Primer sequences of selected genes.

Primer Name	Sequence (5'–3')
p53 Forward	ACCTATGGAAACTACTTCCTG
p53 Reverse	ACCATTGTTCAATATCGTCC
APC Forward	AGAGGTCATCTCAGAACAAG
APC Reverse	CATGTTGATTTCTCCCACTC
KRAS Forward	GGCCTGCTGAAAATGACTG
KRAS Reverse	CTTGCTTCCTGTAGGAATCCTC
c-MYC Forward	TGAGGAGGAACAAGAAGATG
c-MYC Reverse	ATCCAGACTCTGCCTTTTG
PDCD-L1 Forward	GTGGCATCCAAGATACAAACTCAA
PDCD-L1 Reverse	TCCTTCCTCTTGTCACGCTCA
CTLA-4 Forward	TTGCTAAAGAAAAGAAGCCC
CTLA-4 Reverse	AAAGTTAGAATTGCCTCAGC
CD47 Forward	GGCAATGACGAAGGAGGTT
CD47 Reverse	ATCCGGTGGTATGGATGAGA
PTPRC Forward	CTGGATTGACTACAGCAAAG
PTPRC Reverse	GGAAGTATTGTCTGGACTAAG
β-actin Forward	GACGACATGGAGAAAATCTG
β-actin Reverse	ATGATCTGGGTCATCTTCTC
