# Supplementary material for: Gene Expression Analysis of Immune Regulatory Genes in Circulating Tumour Cells and Peripheral Blood Mononuclear Cells in Patients with Colorectal Carcinoma
Source: Int J Mol Sci. 2023 Mar 6;24(5):5051. doi: 10.3390/ijms24055051 (PMC10003441; doi:10.3390/ijms24055051)
Supplement: Supplementary file 1 [file ijms-24-05051-s001.zip › ijms-2200458-supplementary.pdf]

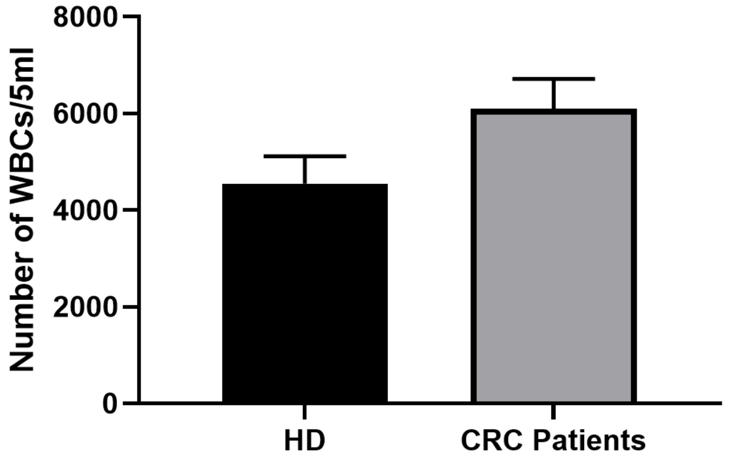

Figure S1: White blood cell (WBC) counts in healthy donors and patients with CRC.

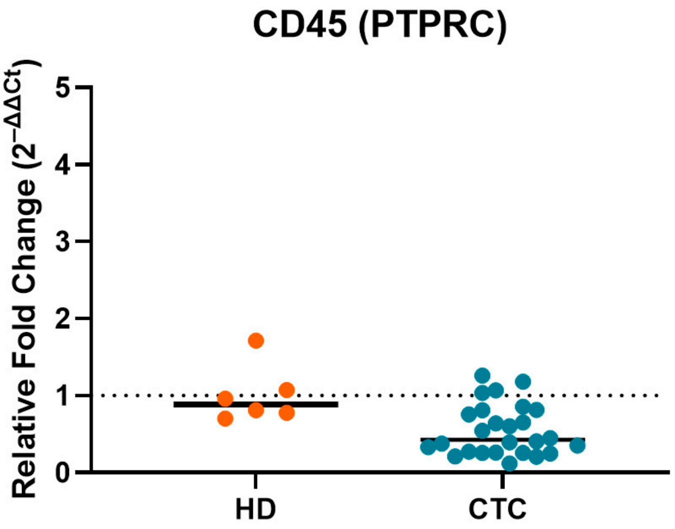

Figure S2: Relative fold change values ($2^{-\Delta\Delta Ct}$) of the *CD45* gene in HDs and in CTC-enriched fractions from patients with CRC.

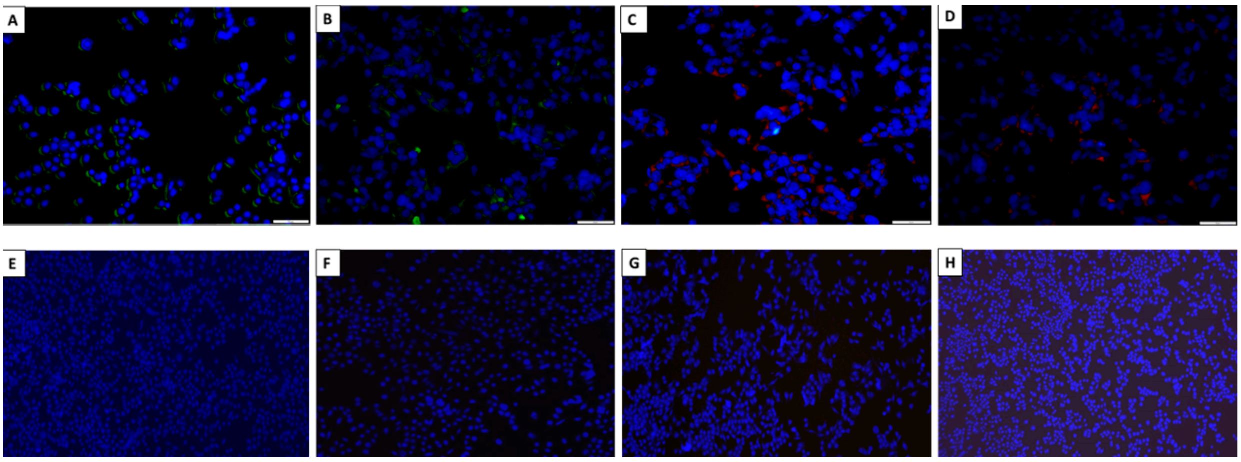

Figure S3: Visualisation of immunofluorescent staining with rabbit anti-mouse (FITC) and rabbit anti-goat (Texas red) to check cross-species secondary binding. (A) to (D) The positive reaction shown with the correct matching of antibodies: staining with (A) mouse anti-Ep CAM and (B) mouse anti-E-cadherin primary, visualised with rabbit anti-mouse (FITC); staining with (C) goat anti-SNAIL1 and (D) goat anti-MMP-9 primary, visualised with rabbit anti-goat (Texas red). (E) to (H) Antibodies are exchanged and there is an absence of staining: staining with (E) goat anti-SNAIL1 and (F) goat anti-MMP-9 primary, and the absence of rabbit anti-mouse (FITC); staining with (G) mouse anti-Ep CAM and (H) mouse anti-E-cadherin primary, and the absence of rabbit anti-goat (Texas red). All the images are merged. Scale bar 50 μm (A, B, C, D); scale bar 10 μm (E, F, G, H).

(**A**)

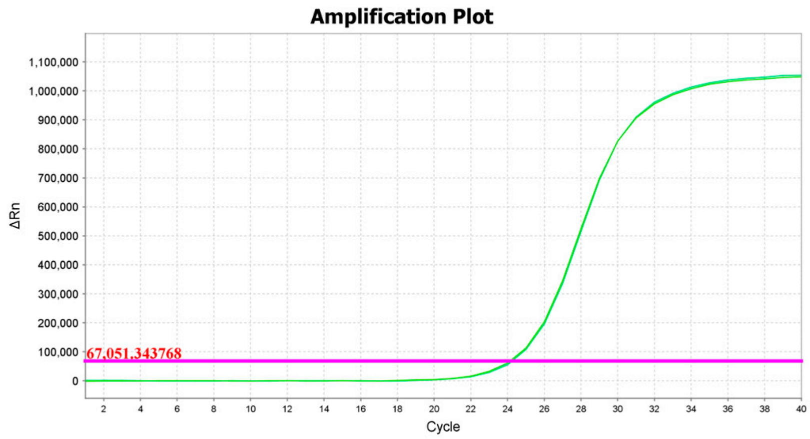

(**B**)

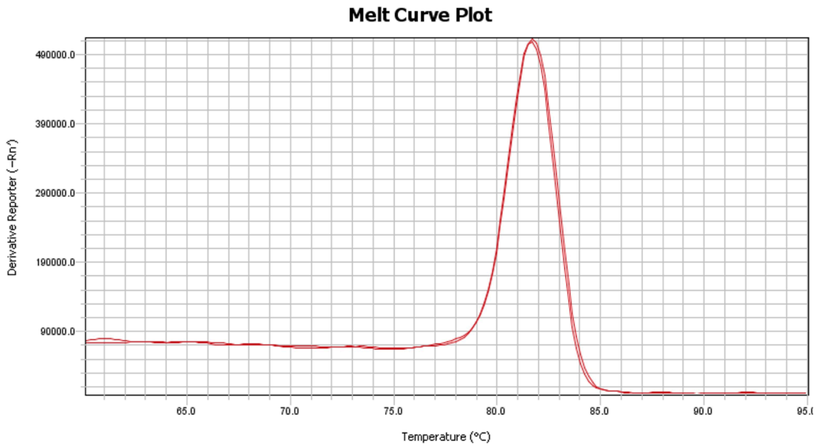

Figure S4: Validation of the Real-Time qPCR Experiment. The RNA integrity extracted from two colon cancer cell lines (SW480, SW48) and one of the CTC and PBMC samples from patients with CRC was evaluated by assessing the gene expression of beta-actin (β-actin). The image showing (A) Ct values of < 30 and (B) a single peak in the melting curve suggesting that the RNA was intact, of good quality and suitable for PCR performance. Table S1: Primer sequences of selected genes.
